# Advancing Cancer Treatment and Diagnosis: A Review on Photodynamic Therapy Using OLED Technology

**DOI:** 10.3390/molecules30061305

**Published:** 2025-03-14

**Authors:** Rajesh Kumar Tiwari, Rajesh Mishra, Sanjay Kumar Sharma, Nakshathra Prabhu, Mangey Ram Nagar, Saulius Grigalevicius

**Affiliations:** 1 School of Information and Communication Technology, Gautam Buddha University, Greater Noida 201312, India; realtiwari@gmail.com (R.K.T.); rmishra@gbu.ac.in (R.M.); sanjay.sharma@gbu.ac.in (S.K.S.); 2 School of Bioengineering and Biosciences, Lovely Professional University, Phagwara 144411, India; naksinmas@gmail.com; 3 School of Electronics, Noida Institute of Engineering and Technology, Greater Noida 201306, India; 4 Department of Polymer Chemistry and Technology, Kaunas University of Technology, Radvilenu Plentas 19, LT50254 Kaunas, Lithuania

**Keywords:** biosensors, NIR-OLED, non-invasive, malignant tumours, organic light emitting diode, photodynamic therapy, photosensitizers, wearable OLEDs

## Abstract

Photodynamic therapy (PDT) is an innovative and non-invasive approach to treating apparent tumours with minimal toxicity. PDT has a long-standing application in antitumor treatment utilizing various photosensitizers (PSs) for different tumours. Historically, light has served as a therapeutic tool in many diseases. PDT involves a dual treatment process in which light energy and PSs are combined to ablate tumour cells following light activation. In general, PDT exhibits reduced side effects and toxicity compared to chemotherapy and radiotherapy, as it spares the extracellular matrix, facilitating excellent tissue healing and minimizing scarring. In addition, PSs can serve in diagnostic roles in tumour identification, termed photodynamic diagnosis (PDD). Advancements in flexible light sources that produce uniform illumination could significantly enhance the consistency of light delivery. This review outlines the clinical applications of OLEDs in PDT for cancer, addressing both diagnostic and therapeutic methods. Furthermore, we will explore various tumour cases using PDT with OLEDs. In particular, antimicrobial PDT targets antibiotic-resistant strains in diabetic foot ulcers, while metronomic PDT promotes cancer cell apoptosis through prolonged, low-intensity light exposure. Our emphasis is on PDT employing organic light-emitting diodes (OLEDs). Furthermore, the combination of PDT with NIR-OLEDs is examined for its potential to enhance tumour-targeting effectiveness, possibly exceeding the results of standalone treatments.

## 1. Introduction

The early diagnosis and treatment of diseases frequently occurs prior to the manifestation of severe symptoms, thereby facilitating the attenuation or cessation of disease progression; consequently, the detection of illnesses in their initial stages provides both effective and economically feasible therapeutic options, thereby increasing the likelihood of patient survival. Diseases that are susceptible to diagnosis and treatment at their nascent stages include various forms of cancer, diabetes [1,2], neonatal jaundice [3], and neural and mental health disorders [4,5]. Cancer represents an extensive array of pathological conditions characterized by the uncontrolled proliferation of abnormal cells within various anatomical regions of the human body. There exists an array of over 100 distinct types of cancer that detrimentally affect the human population [6]. According to the American Cancer Society’s “Facts and Figures 2024”, it is projected that there will be 2,001,140 new cancer diagnoses and 611,720 cancer-related deaths in the United States [7]. The Global Cancer Observatory (GCO), affiliated with the World Health Organization (WHO), re-ports that in 2022, India experienced an incidence of 1,413,316 cancer cases and 916,827 cancer-related fatalities across both genders. Among a demographic of approximately 691,178 males, it has been documented that 15.6% have diagnoses of malignancies in the lip and oral cavity, while 8.5% are affected by lung cancer. Similarly, in a demographic of 722,138 females, 26.6% are diagnosed with breast cancer, and 17.7% are afflicted by cervical cancer. Comparatively, both sexes are significantly impacted by breast cancer (approximately 13.6%), followed by lip or oral cavity cancer (affecting 10.2% of the total population) and cervical or prostate cancer (affecting 9% of the population) [8]. Even today, cervical cancer predominates globally, after breast cancer, particularly among females aged 45 to 55 years. The position paper of the Indian Cancer Society of Cervical Cancer, 2024, notes that the incidence and mortality rate of cervical cancer patients in 2022 remain comparable to those in 2012 [9]. The incontrovertible fact is that there has been no decrease in the rate [9]. Traditional methods for tumour diagnosis include computerized tomography (CT) scans, bone scans, magnetic resonance imaging (MRI), positron emission tomography (PET), ultrasound, X-rays, and biopsy. In contrast, conventional treatment methods comprise surgery, chemotherapy, and radiation therapy, among others. While hybridoma approaches exist for cancer detection and treatment, photodynamic therapy (PDT) and photodynamic diagnosis (PDD) are effective due to their reduced side effects [10]. Photodynamic therapy (PDT) is a therapeutic approach that employs photosensitizers in conjunction with light radiation to eradicate tumour cells. Photodynamic diagnosis (PDD) is a PDT application that leverages the fluorescent properties of the photosensitizers to detect tumour cells. Recent research has indicated that PDT is beneficial in the early stages of cancer diagnosis and treatment globally. However, challenges include limited penetration depth and difficulty in treating large or deep-seated tumours. Light sources used in PDT can include lasers, LEDs, and lamps [11]; when LEDs are replaced with OLEDs, the therapy’s hazardousness is reduced, allowing for extended treatment duration, as OLEDs do not require backlights and are self-emitting sources [12]. This study aims firstly to identify the latest organic materials and their respective wavelengths used in developing effective OLEDs. Secondly, it seeks to highlight the various applications of OLEDs, primarily in detecting and treating diseases, focusing specifically on the treatment of prevalent cancers.

## 2. OLEDs in Healthcare

Organic light-emitting diodes (OLEDs) are increasingly being recognized as prospective light sources for photodynamic therapy (PDT) in oncological treatment and the early detection of critical diseases. OLEDs offer distinct advantages, including flexibility, slim profiles, and area emission, rendering them particularly suitable for wearable and portable PDT devices [13,14]. These properties facilitate prolonged, low-fluence-rate treatments, which have demonstrated potential in addressing gliomas in mouse models [15], healing skin wounds and treating cutaneous leishmaniasis, which can lead to skin cancer lesions [14,16,17,18], detecting and treating ovarian cancer [6,19,20,21], and detecting biomarkers [22], as well as in a drug delivery system model to treat breast cancer [23]. The development of OLEDs for PDT is targeted towards fulfilling specific criteria such as suitable wavelength, light output, uniformity, and durability [24]. Experimental studies have validated the effectiveness of OLED-based PDT in eradicating cancerous cells, bacteria, parasites, and fungi in vitro [15]. The technology exhibits significant potential in overcoming difficulties associated with treating resistant cancers and addressing antimicrobial resistance [24]. As OLED technology progresses, it may revolutionize PDT, making it a more accessible and efficacious therapeutic option for various medical conditions. Recent experiments on glioma mouse models indicate that OLED-based PDT results in extended survival times compared to control groups [15].

**Figure 1:**
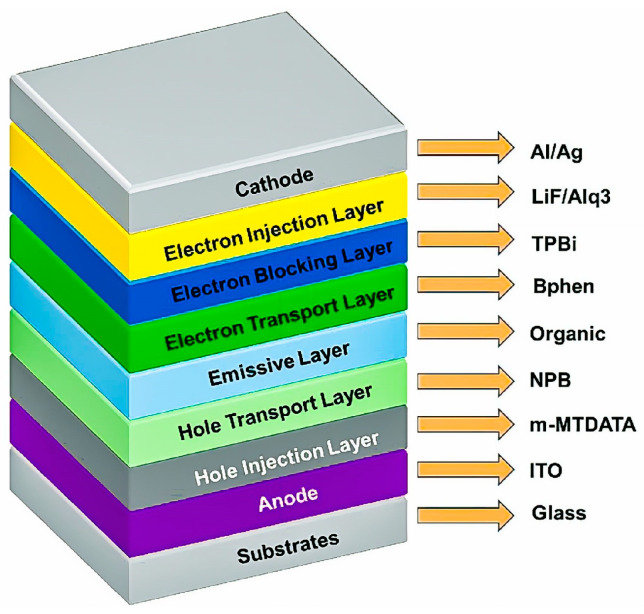
Basic structure and schematic view of multilayered OLED.

In cases of non-melanoma skin cancers, PDT using LEDs has yielded favourable clinical and cosmetic results [25]. Moreover, innovations in nanotechnology, such as quantum dots and liposomes, have further amplified the efficacy of PDT [26]. Additionally, flexible OLEDs have shown effectiveness in antimicrobial PDT, achieving over 99% destruction of Staphylococcus aureus when used in conjunction with methylene blue photosensitizer [27]. OLEDs comprise several thin layers of organic materials positioned between two electrodes: a transparent anode and a metallic cathode. The substrate layer is generally formed from flexible materials such as polyethylene terephthalate (PET) or rigid glass, with preference shown toward flexibility for wearable applications. The anode, commonly made of indium tin oxide (ITO), conducts electricity and permits the transmission of light. The hole transport layer (HTL), constructed from materials such as PEDOT or NPB, facilitates the movement of positive charge carriers to the emissive layer (EML). The EML, often constituted of small molecules like Alq_3_ or polymers such as PPV, is the region where light emission occurs, and its wavelength is contingent upon the employed materials—a crucial aspect for PDT. The electron transport layer (ETL), comprising materials like BPhen or LiF, channels electrons from the cathode to the emissive layer. The cathode is typically fabricated from metals with low work functions, such as aluminium or calcium, to inject electrons into the device. Figure 1 illustrates the layer configuration in an OLED structure [13]. For medical applications such as photodynamic therapy, OLEDs are required to emit specific wavelengths to activate photosensitizers. Advances in phosphorescent materials, like iridium complexes, have enhanced OLED efficiency, while flexible organic semiconductors and nanocomposites improve durability. Near-infrared (NIR) OLEDs prove particularly effective in treating tumours located deeper within tissues. These technological advancements render OLEDs ideal for continuous treatments in conditions such as gliomas and in antimicrobial therapy.

## 3. Photodynamic Diagnosis and Treatment

The term denotes the employment of light photons in medical diagnostics and therapies. Biochips and biosensors have been developed to fulfil the objective of disease diagnosis [6,28,29], whereas, for therapeutic interventions, photodynamic therapy (PDT) is favoured, whether applied invasively or non-invasively. This technique utilizes light energy in conjunction with photosensitizers to target and destroy malignant cells. A photosensitizer functions as a light-sensitive catalyst that, upon absorbing light energy at a specific wavelength, excites and transfers energy to molecular oxygen in the surrounding environment, generating reactive oxygen species (ROS), including the singlet O_2_ and other radicals, which culminate in a cytotoxic impact leading to the destruction of tumour cells [30]. Due to its high reactivity, singlet O_2_ can inflict severe damage on various cellular components. This occurs through the direct disruption of cellular structures such as membranes, mitochondria, lysosomes, and nuclei via mechanisms like apoptosis, necrosis, and autophagy, or indirectly by inciting a subsequent oxidative stress surge that aggravates the initial damage and affects neighbouring cells and tissues. ROS can impair the PS, rendering it non-fluorescent [10,23]. The use of diverse photosensitizers for cancer diagnostics is generally termed photodynamic diagnosis (PDD), where the fluorescence emitted by PS is employed to detect cancerous tissues [11]. By increasing the illumination intensity or duration of PDD, it transitions into PDT [10]. An innovation in PDT is metronomic PDT, which utilizes a lower dosage of PS and a reduced light source intensity, thus necessitating more frequent irradiation. Photosensitizers (PS) utilized in photodynamic therapy (PDT) are categorized into three generations. First-generation PSs, such as Porfimer sodium (Photofrin), have been sanctioned for cancer therapies but present limitations, including extended skin photosensitivity. Second-generation PSs, such as mTHPC (Temoporfin) and 5-aminolevulinic acid (5-ALA), demonstrate improved tumour specificity, better tissue penetration, and diminished skin sensitivity. Third-generation PSs are coupled with targeting agents like antibodies or nanoparticles to enhance specificity towards cancer cells, as exemplified by Photofrin conjugated to monoclonal antibodies [10]. PDT is employed in the treatment of various cancers, including those affecting the skin, head and neck, lung, oesophagus, ovaries, breast, and prostate. For skin cancers, 5-ALA and methyl amino levulinate (MAL) are effective in non-melanoma cases. Porfimer sodium finds extensive application in head and neck, lung, and oesophageal cancers. When combined with advanced delivery systems such as nanoparticles, PDT holds potential for breast and ovarian cancer management. Research in prostate cancer emphasizes the effectiveness of second-generation PSs like Tookad soluble. Future advancements in PDT prioritize the integration of nanotechnology and targeted delivery mechanisms to enhance PS selectivity while minimizing collateral damage to healthy tissues. Innovations like near-infrared (NIR) light and metronomic PDT (mPDT) propose efficient treatment modalities with reduced adverse effects [11]. Important parameters for PDT light sources include power density, typically ranging from 10 to 200 mW/cm^2^ depending on the treatment type, radiation stability to ensure reproducible photon output over prolonged exposure, and service life, often exceeding 10,000 h for high-quality OLED-based PDT sources. These parameters are instrumental in providing reproducible and efficient therapeutic efficacy [16].

### 3.1. Ovarian and Prostate Cancer

Early identification of tumours is correlated with improved outcomes for patients. Ovarian cancer, which had a global incidence rate of 4.0 ASR per 100,000 individuals in 2022, poses significant challenges for detection, thereby being a focal point for research due to the predominance of late-stage diagnoses. As a result, an OLED-based, non-invasive, portable sensor has been devised to aid early detection, a critical factor for improving survival rates [6]. A triple-hole block-layer OLED has been constructed to exhibit enhanced luminescence and efficiency compared to traditional multilayered OLEDs [19]. This device has shown potential for the diagnosis of ovarian cancer by identifying fluorescence in urine samples at specific wavelengths. In a preceding study, the multilayered and triple-hole block-layer OLEDs were evaluated for their effectiveness in cancer detection [31]. While multilayered OLEDs are deemed superior in terms of light detection, triple-hole block-layer OLEDs excel as light sources. The researchers also integrated a dual-gate organic thin-film transistor to drive the OLED light source. Both investigations underscore the potential of OLED-based sensors to develop portable, flexible, and cost-effective biomedical devices for ovarian cancer screening. The suggested methods depend on discerning differences in fluorescence between healthy individuals and cancer patients at specific wavelengths of light. The device can autonomously differentiate between healthy individuals and ovarian cancer patients based on the fluorescence of their urine at specific wavelengths. Samples from cancer-affected patients exhibit higher fluorescence, whereas samples from healthy patients demonstrate lower fluorescence. The proposed methodology strives to establish a low-cost, user-friendly biomedical sensor for ovarian cancer diagnosis at home. Urine analysis is also favoured for other gynaecological cancers and bladder cancer determinations [32], owing to the presence of various intrinsic fluorophores in human urine; employing advanced fluorescence techniques, diseases can be tested and diagnosed [6,19].

**Figure 2:**
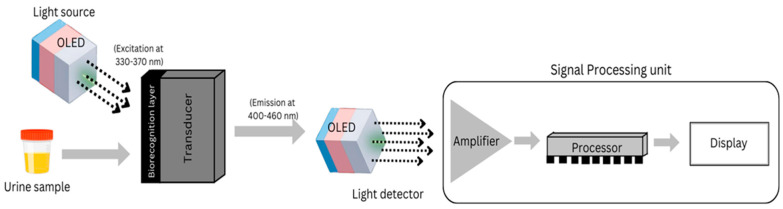
Mechanism of detection of ovarian cancer using two OLEDs, one as a light source to excite rays on the urine sample collected from the individuals and another as a light detector.

Figure 2 illustrates the detection mechanism for ovarian cancer, utilizing two OLEDs: one as a light source emitting rays in the 300–400 nm range on the urine sample collected from individuals, and another as a light detector to measure the amount of emitted rays from the urine sample. This allows differentiation between cancerous and healthy patients based on fluorescence intensity (I_f_), which is computed using the Beer–Lambert law:

I_f_ = ϕ.ϵ.l.C.I_o_ (1)

Here, ‘ϕ’ and ‘ϵ’ refer to the quantum yield of the fluorophores and molar absorptivity of biomarkers, respectively, while ‘l’ and ‘C’ are the path length of the light in the sample and the concentration of fluorescence biomarker. ‘I_o_’ is the intensity of excitation light. The initial phase in employing OLEDs for light detection involves calculating its emission wavelength via the following equation:

E_g_ = hν = hc ÷ λ (2)

where ‘E_g_’ denotes the band gap of the light-emitting substance, ‘h’ signifies Planck’s constant, ‘c’ represents the speed of light, and ‘λ’ indicates the emitted light’s wavelength. There is a shift in the wavelength, which is determined by the following:

∆λ = λ_2_ − λ_1_ (3)

In this context, ‘∆λ’ is the Stokes shift, whereas ‘λ_1_’ and ‘λ_2_’ are wavelength of excitation and emission, respectively. The binding kinetics of biomarkers to the biorecognition layer can be described by the Langmuir adsorption isotherm, defined as follows:

θ = [B] ÷ (K_d_ + [B]) (4)

where ‘θ’ represents the fraction of occupied binding sites, ‘[B]’ denotes the biomarker concentration, and ‘K_d_’ indicates the dissociation constant reflecting the biomarker’s affinity for the recognition element. The final output (V_out_) is determined by the following:

V_out_ = G.I_f_ (5)

where ‘G’ is the gain of amplifier and ‘I_f_ ’ is the fluorescence intensity. To detect whether the sample contains cancerous biomarkers, I_f_ is compared with I_threshold_. If I_f_ > I_threshold_, then it is cancerous. In the advancement of ovarian cancer treatment, photodynamic therapy (PDT) is increasingly recognized as a promising modality, notwithstanding its traditionally poor prognosis and associated high mortality rates [20,30]. PDT involves the use of photosensitizers that generate cytotoxic reactive species upon exposure to light, resulting in targeted cell death. To enhance the selectivity of treatment and reduce toxicity, research focuses on nanoparticle-based delivery systems for photosensitizers and targeted photo-immunoconjugates. Such strategies seek to address the challenges of chemoresistance and non-specific toxicity that are prevalent in traditional PDT [20]. Importantly, biodegradable nanoparticles have shown enhanced cytotoxic effects in ovarian cancer cells when used in conjunction with near-infrared light, indicating a potential improvement in treatment efficacy through the use of targeted multimodal therapies [21,33]. This stream has more scope for research in the future, which is unnoticed. Table 1 reviews literature discussing the diagnosis and treatment of ovarian cancer using OLED technology, particularly highlighting the superior luminescence and efficiency of the triple-hole block-layer OLED compared to traditional multilayer OLEDs, with TADF OLEDs demonstrating even greater efficiency, which is vital for fluorescence detection of biomarkers in the 300–400 nm emission range essential for early ovarian cancer diagnosis.

### 3.2. Cutaneous Tumors and Wound Healing

Cutaneous tumours represent a heterogeneous group of skin lesions, categorized into benign or malignant forms. Research has indicated a higher prevalence of benign tumours, notably seborrheic keratosis, compared to their malignant counterparts [34,35,36,37,38]. Benign tumours may originate from a variety of skin structures, including the epidermis, melanocytes, and connective tissues. In contrast, malignant tumours, comprising melanoma, squamous cell carcinoma, and basal cell carcinoma, although less frequent, pose a greater health concern due to their potential to cause significant morbidity [39]. Cutaneous tumours exhibit a range of clinical features, with distinguishing characteristics between benign and malignant types. Parameters such as age, gender, and lesion morphology can assist in differentiating between these tumour categories. The utilization of non-invasive screening instruments, particularly those employing LED technology, facilitates the early detection of skin cancer and the assessment of postoperative scars [40]. Optical Coherence Tomography (OCT) offers valuable morphological and angiographic data, augmenting the sensitivity and specificity of melanoma detection through advanced image processing and machine learning applications [41]. The early diagnosis and effective management of cutaneous melanoma are pivotal in improving survival rates, underscoring the importance of prompt detection for successful treatment. Innovative antibody-based strategies, especially monoclonal antibodies targeting tumor-associated antigens, are emerging as potent diagnostic and therapeutic modalities. Antibody–drug conjugates (ADCs) incorporating non-toxic photo-activatable photosensitizers enable targeted photodynamic therapy, allowing the selective eradication of cancer cells while sparing normal cells. Recent advancements in antibody-mediated interventions offer novel possibilities for enhancing and complementing existing methodologies for the diagnosis and treatment of skin cancer [42]. Photodynamic therapy (PDT) is emerging as an effective non-invasive therapeutic option for various dermatological conditions, particularly non-melanoma skin cancers. It has demonstrated efficacy in the treatment of actinic keratoses, basal cell carcinomas, and Bowen’s disease [43,44]. For cutaneous squamous cell carcinoma in situ, the effectiveness of PDT is influenced by variables such as tumour location, size, and photosensitizer incubation time [45]. Nevertheless, its efficacy in treating invasive squamous cell carcinoma is compromised due to inadequate penetration of photosensitizers through keratinized tumour surfaces [46].

To enhance PDT efficacy, several strategies have been investigated, including pretreatment with lasers or microneedles and nanocarrier-based delivery systems for photosensitizers [46,47]. These nanocarriers aim to improve the tissue penetration and stability of photosensitizers, potentially broadening PDT applications within dermatology [47]. Despite challenges such as procedural pain and treatment costs, PDT is increasingly regarded as viable for managing a range of dermatological conditions, including inflammatory and infectious diseases [44]. Photodynamic therapy (PDT) in conjunction with Mohs micrographic surgery (MMS) demonstrates potential in the treatment of basal cell carcinoma (BCC) and squamous cell carcinoma (SCC). Research suggests that PDT followed by MMS may decrease the number of surgical stages and reduce the final defect size com-pared to MMS alone for BCC [48]. A retrospective study identified no significant difference in recurrence rates between PDT combined with surgery and MMS for challenging cases of BCC. However, the healing period was extended with the combined approach [49].

For early-stage oral SCC, PDT exhibited comparable efficacy to surgical resection regarding complete response and recurrence rates [50]. Moreover, MMS followed by topical PDT for facial BCC yielded low recurrence rates and preserved tissue integrity [51]. While these findings are encouraging, there is a necessity for larger, multi-centre trials to further evaluate the effectiveness of combining PDT with MMS for various skin cancers. OLEDs have exhibited efficacy in antimicrobial photodynamic therapy for the treatment of cutaneous leishmaniasis, with 1,9-dimethylmethylene blue identified as an effective photosensitizer [34,36]. The flexibility and thinness of OLEDs render them suitable for wearable phototherapeutic devices, as evidenced by a study that developed a 6 µm thick OLED-based photonic skin that enhanced artificial skin regeneration by up to 70% [35]. These results imply that OLEDs hold substantial potential as light sources for diverse phototherapeutic applications, providing advantages such as consistent emission, compactness, and the capability to create conformable, skin-like platforms for treatment. Organic light-emitting diodes (OLEDs) constitute an innovative light source appropriate for photobiomodulation therapy (PBMT) aimed at wound healing and skin rejuvenation, without inducing skin burns. OLEDs have demonstrated comparable efficacy to traditional light-emitting diodes (LEDs) in accelerating wound healing by modulating cytokine synthesis and growth factor gene expression [4]. The intrinsic benefits of OLEDs, such as uniform irradiation, flexibility, and minimal heat production, make them ideal candidates for wearable PBMT devices [37]. The PCBA within the device regulates OLED operations, enabling customization of radiance duration in minute and second settings and current settings from 0 to 35 mA. The OLED is encapsulated to prevent external damage, and a 3M Tegaderm film protects against wound exudate, also providing a moist environment and facilitating 80% high transmittance of light [52]. Fluorescence at 633 nm has been shown to aid cell proliferation, enhancing wound healing. Both in vitro and in vivo studies have demonstrated that OLED irradiation can increase collagen expression, reduce matrix metalloproteinase levels, and promote growth factor expression in human dermal fibroblasts. Moreover, OLED exposure has been shown to mitigate skin wrinkles and augment dermal collagen density in UV-irradiated animal models [4]. These insights suggest that OLED-based wearable devices possess the potential to revolutionize dermatological treatments and wound-healing therapies [18]. Moreover, studies have demonstrated that OLED irradiation can expedite diabetic wound healing in rats by enhancing fibroblast growth factor-2 expression and augmenting macrophage activation [53]. Table 2 reviews the literature discussing the diagnosis and treatment of skin cancers and wound healing utilizing wearable OLEDs.

### 3.3. Glioma/Brain Injury

Photodynamic diagnosis (PDD) and photodynamic therapy (PDT) are effective techniques for managing malignant gliomas, which are challenging to treat due to their invasive nature [54]. PDD utilizes fluorescent dyes to improve tumour visualization during surgery [54,55], while PDT involves administering a photosensitizer followed by light activation to target tumour cells [56]. Optical techniques can enhance biopsy sampling safety and efficacy while also monitoring PDT effectiveness [55].

Ongoing research aims to develop clinically predictive models for glioma treatment using PDT. Despite its potential, further studies are needed to fully understand PDT’s immunomodulatory effects and its efficacy in combination with other therapies for high-grade gliomas [56]. Photodynamic therapy (PDT) shows promise as a treatment for glioblastoma, the most common and aggressive adult brain cancer, and other cancers in resource-limited settings [57,58]. PDT involves administering a photosensitizer, which is activated by specific wavelengths of light to produce reactive oxygen species that induce tumour cell death [57,59]. The most commonly used photosensitizer is 5-aminolevulinic acid (5-ALA), which is metabolized selectively in cancer cells. Despite its potential, PDT faces challenges in clinical translation, including variable uptake of photosensitizers, insufficient tissue penetrance of light, and poor oxygen recovery in tumours. Researchers are exploring novel approaches to improve PDT’s efficacy, such as nanoparticle-linked photosensitizers, photoimmunotherapy, and near-infrared radiation [57]. Additionally, PDT has shown promise in opening the blood–brain barrier, which could enhance drug delivery to brain tumours [60]. Low-cost LED-based devices can effectively deliver light for PDT, even when powered by disposable batteries [61]. Studies have investigated various photosensitizers, including curcumin, methyl amino levulinate, and 5-aminolevulinic acid (5-ALA), which can cross the blood–brain barrier [62,63]. Researchers have explored optimal light doses and fluence rates for different glioblastoma subtypes to enhance treatment efficacy [63]. Additionally, optical techniques can improve the diagnosis and monitoring of glioblastoma during treatment [55]. Recent advancements in organic electronics have led to the development of organic light-emitting diodes (OLEDs) and organic photodetectors (OPDs) for lab-on-a-chip bio-detection systems [52]. These devices offer the potential for miniaturized, low-cost, and disposable test arrays. Ultra-flexible OLEDs have been successfully used for optogenetic nerve stimulation in rats [64]. Semi-transparent OLEDs have also been applied to optical brain imaging. Furthermore, OLEDs have shown promise in photodynamic therapy for treating gliomas in mouse models [15]. These studies highlight the versatility and potential of OLEDs in various biomedical applications, from biosensing to neuronal stimulation and cancer treatment. Table 3 describes the papers that discuss the diagnosis and treatment of gliomas and mental health issues such as brain injuries using OLED.

### 3.4. Breast Cancer

Breast cancer is a prevalent and life-threatening disease among women, necessitating early detection for improved survival rates [66]. While traditional diagnostic methods like mammography and biopsies exist, they have limitations such as high costs and invasiveness [67].

PDT has traditionally been used for surface treatments; it involves using photosensitizers, light, and oxygen to generate cytotoxic reactive oxygen species, particularly singlet oxygen, which leads to tumour cell death [68,69]. Recent advancements are exploring its potential for deep-seated cancers like breast cancer [70], the most common cancer in women worldwide [71]. One innovative approach is bioluminescence-induced proteinaceous PDT (BLiP-PDT), which combines luciferase and reactive oxygen species-generating protein to create a self-luminescent, degradable system that doesn’t require external light irradiation [72]. This method has shown promising results in primary breast cancer cells and in vivo tumour xenograft models. Despite these advancements, challenges remain, such as improving light delivery to deep tissues and developing more effective photo-sensitizers [68,69]. To overcome the limitations of conventional PS, such as poor water solubility and non-specificity, researchers have developed nanoparticle-based delivery systems. Metal nanoparticles offer advantages like high stability, adjustable size, and easy surface functionalization, while organic nanoparticles can be functionalized for active targeting. These nanocarriers improve PS biodistribution and tumour uptake, enhancing PDT efficacy. Targeted PDT using nanoparticles shows promise as a non-invasive, site-specific treatment for breast cancer [71,73]. Biomarkers play a crucial role in early cancer detection and treatment evaluation. However, relying on single biomarkers like CEA is insufficient, leading to the need for effective biomarker combinations [66]. Biosensor-based diagnostic approaches have emerged as promising alternatives, offering advantages such as cost-effectiveness, non-invasiveness, and high through-put. These biosensors can detect various genomic, transcriptomic, proteomic, and metabolomic biomarkers associated with breast cancer. The development of biosensor-based technologies for breast cancer biomarker detection could potentially reduce the economic burden and improve accessibility to early diagnosis, especially in underserved populations [67]. A novel self-powered wireless device for metronomic photodynamic therapy (PDT) in cancer treatment consists of a semi-invasive pedestal with a micro-LED and syringe needle and a detachable actuator containing a drug reservoir, thermally driven pump, and wireless control circuit. It can deliver on-demand dosages of drugs and light into tumours beneath the skin. The device can be partially self-powered using body motion energy through piezoelectric nanogenerators. In vitro and in vivo experiments on mice demonstrated the device’s effectiveness, with tumour volumes in the treatment group reduced by 57.4% compared to the control group. This innovative approach offers a flexible and convenient strategy for low-dose delivery of light and drugs, potentially replacing invasive optical fibres in PDT. The researchers suggest that this device has promising applications in future clinical cancer treatment and expands the scope of self-powered systems in biomedical engineering [23]. Table 4 evaluates the papers that discuss the diagnosis and treatment of breast cancer using OLEDs.

### 3.5. Detection of Biomarkers

Biosensor technology has shown promise for early cancer detection through the identification of specific biomarkers. Optical and photo electrochemical Nano biosensors offer high sensitivity and specificity in detecting cancer biomarkers at low concentrations [22]. These biosensors utilize various transduction systems, including electrochemical, optical, and mass-based approaches, to detect nucleic acid and protein-based cancer biomarkers [75].

Additionally, photodynamic therapy (PDT) has emerged as an effective, minimally invasive treatment for cancer. Recent developments in targeted PDT have focused on delivering photosensitizers to specific cancer biomarkers, improving treatment efficacy and reducing toxicity to normal tissues [76]. These advancements in biosensor technology and targeted PDT offer promising avenues for early cancer diagnosis and improved treatment outcomes. The latest improvements in photonic crystal-based biosensors have shown promise for detecting various cancer biomarkers, including circulating tumour DNA, miRNA, proteins, and metabolites.

These sensors, utilizing 1D, 2D, and 3D configurations, offer improved electromagnetic confinement and detection limits [77]. Complementing this approach, disposable point-of-use optical biosensors using organic light-emitting diode (OLED) technology have been developed for multiple biomarker detection. While initial designs required optical filters to achieve sensitivity comparable to laboratory instruments [4], further refinements have led to the integration of highly flexible OLED display technology with protein microarray technology. The proposed method aims to significantly reduce the cost of pre-functionalized biosensor substrates to pennies per square centimetre by leveraging commercial OLED display technology. This combination enables high-density, programmable, and multiplexed biorecognition in a compact, disposable format with clinical-level sensitivity. This level of sensitivity is comparable to the lower limit of detection typically found in clinical laboratory instrumentation. The approach has demonstrated quantitative detection of IgG antibodies to viral antigens and HPV16 proteins, which are biomarkers for cervical, head, and neck cancers, with detection limits as low as 10 pg/mL. The innovative combination of OLED technology with immunobiosensors has the potential to revolutionize point-of-care diagnostics by offering high-performance, low-cost solutions that can be mass-produced using existing display manufacturing capabilities [78]. Another development of a miniaturized biochip utilizes a novel deep-blue organic light-emitting diode (OLED) for protein microarray fluorescence detection. The researchers designed and optimized the molecular structure of the OLED to effectively excite fluorophore-conjugated antibodies. The biochip was tested in a protein microarray configuration, demonstrating good sensitivity and specificity. The integration of OLEDs in biochips offers several advantages for point-of-care medical diagnostic systems, including portability, cost-effectiveness, and broader accessibility to healthcare worldwide. By combining multidisciplinary approaches, these integrated biochips aim to decouple diagnosis from centralized laboratories. The findings of this study are expected to contribute significantly to the development of next-generation point-of-care biochips, potentially revolutionizing medical diagnostics by making them more accessible and efficient [28]. Table 5 evaluates the papers that discuss the detection of biomarkers using OLEDs, which leads to further analysis and treatment of tumours.

### 3.6. Neonatal Jaundice

Neonatal jaundice, affecting up to 80% of preterm infants, requires careful monitoring and treatment to prevent severe complications [79]. Traditionally, neonatal jaundice is treated in incubators, where the neonate is hospitalized for days and a range of 430–490 nm blue spectrum beams of rays are passed after covering the eyes of the baby. This may cause retinal damage, dehydration, and separation from their parents [3]. Phototherapy is a widely used, non-invasive treatment for neonatal jaundice, effective in reducing serum bilirubin levels [81]. It works by transforming native bilirubin into water-soluble photo isomers that can be excreted [82,83]. While generally safe, phototherapy may have side effects, including hyperthermia, skin rashes, and dehydration in preterm infants. Recent studies suggest that aggressive phototherapy in extremely low birth weight infants may increase mortality risk, possibly due to reduced bilirubin’s antioxidant effects [83].

However, phototherapy remains the primary treatment for unconjugated hyperbilirubinemia in both term and preterm infants [84]. Ongoing research continues to refine our understanding of phototherapy’s mechanisms and optimal application in neonatal care. Recent studies have explored the efficacy of different light sources for treating neonatal jaundice. Blue-green light with a wavelength of 460–490 nm is most effective, with LED sources now preferred over fluorescent or halogen lights. Ebbesen et al. have compared turquoise and blue LED lights, finding no significant difference in their bilirubin-reducing effects [82]. Savedra et al. have demonstrated that white LED phototherapy promotes more efficient bilirubin degradation than conventional blue-light therapy in vitro [85]. Sherbiny et al. have conducted a randomized trial comparing high-intensity LED beds with conventional fluorescent tube units for intensive phototherapy. They reported significantly higher success rates and bilirubin decline rates with the LED treatment, particularly in both haemolytic and non-haemolytic subgroups. Additionally, the LED group experienced fewer side effects such as hyperthermia, dehydration, and skin rash compared to the fluorescent tube group. These findings suggest that LED-based phototherapy, especially high-intensity or white LED, may offer improved treatment options for neonatal jaundice, potentially reducing the need for exchange transfusion [86]. Recent advancements in phototherapy include the development of wearable devices using blue organic light-emitting diodes (OLEDs). These textile-based platforms offer flexibility, conforming to the body’s curvature as an alternative to traditional LED arrays, providing effective treatment at low voltages with peak wavelengths of 470 nm. This textile-based OLED system demonstrated performance appropriate for intensive treatment at low voltages, with over 100 h of operational reliability and low-temperature operation. In vitro tests demonstrate the ability of OLED platforms to reduce bilirubin levels significantly within hours of irradiation. The textile-OLED platform offers advantages over conventional LED arrays, including flexibility, uniform treatment, and potential for improved patient comfort. Additionally, textile-based OLEDs have shown promise for fashion displays and exhibit washability, further expanding their potential applications in wearable technology [3]. Additionally, innovative wearable sensors have been created for real-time, colorimetry-based jaundice detection, simultaneously measuring oxygen saturation and heart rate [87]. These devices can function during phototherapy, potentially enabling automated, optimized treatment approaches. While various phototherapy methods exist, further research is needed to determine optimal wavelengths, intensities, and dosages for different patient populations [84]. Table 6 assesses the papers that discuss the diagnosis and treatment of neonatal jaundice using blue-OLED as a wearable textile.

## 4. Near-IR OLED

Photodynamic therapy (PDT) has focused on developing near-infrared (NIR)-absorbing organic nanoparticles for targeted cancer treatment. These nanoparticles offer improved tissue penetration depth and biocompatibility compared to traditional photosensitizers [89]. A novel approach for wireless deep-tissue photodynamic therapy (PDT) uses a flexible implant containing up conversion nanoparticles (UCNPs) [90]. The implant consists of a biocompatible poly(ethylene glycol) diacrylate (PEGDA) core encased in fluorinated ethylene propylene (FEP), addressing concerns about UCNP retention in tissues. The device can convert near-infrared (NIR) light to visible light, which activates the photosensitizer 5-aminolevulinic acid (5-ALA). The implant can transmit upconverted light up to 8 cm in length, even when bent or implanted under the skin or scalp. The researchers demonstrated the efficacy of this system for chronic PDT in a mouse model of glioblastoma multiforme (GBM). This approach represents a significant advancement in wireless deep-tissue phototherapy, potentially improving clinical translatability by allowing UCNP sequestration while maintaining effective light delivery for PDT applications. Carbazole-substituted BOD-IPY molecules have shown promise, with high singlet oxygen quantum yields and broad NIR absorption [90]. Resonance energy transfer mechanisms have been employed to enhance NIR photon utilization and singlet oxygen generation. Pyrrole-substituted iodinated BODIPY molecules encapsulated in polypeptide nanoparticles have demonstrated high singlet oxygen yields and effective tumour targeting. These nanoparticles can be activated using low-power NIR light sources, including cost-effective lamp lights and LEDs, making them suitable for deep-tissue tumour targeting. Additionally, their NIR fluorescence properties enable simultaneous in vivo tracing, offering the potential for precise tumor-targeting theragnostic and affordable clinical cancer treatments [91,92,93]. NIR photoimmunotherapy (NIR-PIT) utilizes monoclonal antibodies conjugated to photoactive agents for selective cancer cell destruction. Diagnostic tools like diffuse correlation spectroscopy (DCS) and NIR spectroscopy (NIRS) enable tumour detection by observing erythrocyte movements and tissue chromophore differences [94]. Diffuse optical tomography, a cost-effective NIR-based imaging method, offers non-invasive cancer screening for soft tissues [95]. Additionally, wearable and wavelength-tunable NIR organic light-emitting diodes (OLEDs) have been developed for phototherapeutic applications, demonstrating enhanced cell-proliferation effects compared to visible light irradiation. These OLEDs can emit wavelengths from 700 to 800 nm and maintain stable operation under bending conditions, making them suitable for wearable biomedical applications [96]. These advancements highlight the potential of NIR technology in improving cancer diagnosis and treatment methods. Near-infrared (NIR) light has emerged as a powerful tool for precise diagnosis and therapy of cancer. NIR bioimaging offers high sensitivity and resolution for bone cancer detection and intraoperative visualization [97]. NIR photoimmunotherapy (PIT) combines targeted antibodies with photosensitizers to induce rapid and selective cancer cell death upon NIR light exposure [98]. This approach shows promise for treating breast cancer, offering an alternative to conventional therapies [68]. Recent advances include the development of novel fluorescent probes, such as lycosin-I peptide-modified gold nanoclusters for cancer cell imaging and semiconducting polymer nanoparticles for NIR-II fluorescence imaging-guided photothermal therapy [99]. These technologies leverage the advantages of NIR light, including reduced tissue scattering and absorption, to enhance imaging resolution, signal-to-noise ratio, and penetration depth. Ongoing research aims to overcome current limitations and expand clinical applications of NIR-based cancer diagnosis and treatment. Microcavity tandem near-infrared (NIR) organic light-emitting diode (OLED) possesses improved performance characteristics, including narrow full-width half-maximum, high radiant emittance, and external quantum efficiency. The researchers investigated the cell-proliferation effects of OLEDs across the visible and NIR spectrum on human fibroblasts, marking the first comprehensive analysis of this kind using OLEDs. The results revealed that certain wavelengths promoted cell proliferation, with the overall trend mirroring the cytochrome C oxidase absorption spectrum of human fibroblasts [100]. The therapeutic window size for PDT application in NIR is typically between 600 nm and 850 nm for non-superficial treatment. In superficial treatment, where generally blue (~400 nm) is used, has a penetration depth around 1–2 mm, while in the case of NIR, it varies up to maximum 1 cm [16]. For NIR application, more than 850 nm is not advisable as the penetration depth decreases. The average penetration depth between 650 nm and 750 nm is around 5 mm (i.e., up to Subcutis), and around 1000 nm it reduces to 2 mm (i.e., up to Dermis) [16,94]. The design of a customized device for NIR-specific wavelength depends upon the type of cancer and material selection. This research highlights the potential of OLEDs as a next-generation light source for biomedical applications, particularly in therapies requiring deep tissue penetration. This study’s findings contribute to the growing body of knowledge on the therapeutic effects of light-emitting devices and pave the way for future investigations into OLED-based medical treatments. Many of the application as suggested in Table 7 shows the application of NIR OLEDs in diagnosis and treatment of various diseases.

## 5. Comprehensive Analysis

Factors affecting the OLED-PDT are also governed by the type of technology and generations. The emissive layer material of the OLED decides the quality and overall performance of the OLED being used in the PDT. This is summarized in Table 8.

Some studies report sensitivities in terms of nanometre per refractive index unit (nm/RIU) range for addressing an optical domain to represent peak detection, while some of the studies show the sensitivity limits based on biomarkers. In terms of optical sensitivity, the average sensitivity of peak detection is in the range of 100 nm/RIU for detecting specific cancer proteins for OLED-PDT devices. The limit of detection (LOD) is a key metric for sensitivity, and the review of the literature shown in Table 9 shows some of the biomarkers and their average detection limits. Specificity is also crucial, which ensures that the biosensor detects the intended cancer biomarker only and no other artefacts affect it.

## 6. Conclusions

In conclusion, the integration of organic light emitting diodes (OLEDs) into photodynamic therapy (PDT) represents a significant advancement in the field of cancer treatment and diagnostics. This review has highlighted the multifaceted applications of OLEDs, emphasizing their potential to enhance the efficacy of PDT in various cancer types, including ovarian, prostate, skin, glioma, and breast cancers. The unique properties of OLEDs, such as flexibility, low power requirements, and the ability to emit specific wavelengths, make them particularly suitable for non-invasive therapeutic and diagnostic applications. Furthermore, the combination of OLEDs with innovative techniques such as metronomic PDT and near-infrared (NIR) light therapy has shown promise in overcoming traditional limitations associated with photosensitizers and light penetration in deeper tissues. As research continues to refine these technologies, OLEDs may revolutionize the landscape of cancer treatment, offering safer, more effective, and accessible options for patients worldwide. Future studies will be essential to address existing challenges, optimize treatment protocols, and fully realize the potential of OLED-based PDT in clinical settings.

## Figures and Tables

**Table 1:** Literature review of diagnosis and treatment of ovarian cancer using OLEDs.

S.No	Ref.	Source and Year of Publication	Advantages	Limitations
1.	[6]	2018 IEEE	- A home-useable gadget that is lightweight and unobtrusive for early detection. - User-friendly and early detection enhances the survival rate.	Not mentioned.
2.	[31]	2019 IEEE	- Robust, lightweight, and low power requirements. Superior light detection capabilities. - The ability of the dual-gate OTFT indual-gate mode to generate the 18 V required to operate the triple-hole block-layer OLED light source.	- An immobile, non-flexible ovarian cancer screening and diagnosis tool. - Limit its ability to detect a wider range of wavelengths.
3.	[19]	2021 IEEE	- An increase in luminous power efficiency of 74%. - Improved recombination rates. - Good response to varying wavelengths with a maximum photocurrent of 93 mA. - The capacity to distinguish between healthy people and those who have ovarian cancer using the cathode current and fluorescence that urine samples produce.	Not mentioned.
4.	[20]	2019 MDPI	- It can overcome challenges associated with ovarian cancer, including chemoresistance. It can resensitize ovarian tumours to chemotherapy, increasing treatment options. - Allows combination therapy and dose reduction of toxic drugs. - It has the potential to improve patient quality of life.	- Non-specific localization of photosen sitizers leads to dose-limiting toxicities. - Lower effective concentration of photo sensitizers delivered to target cells with PICs compared to free photosensitizers. - Limited light penetration depth.
5.	[21]	2020 Elsevier	- Increased stability and loading efficiency of the photosensitizers. - Improved delivery capacity and stronger quantum yield/phototoxicity. - Overcoming the lack of tumour specificity of photosensitizers.	- Tumour heterogeneity. - Lack of specificity of photosensitizers for tumours. - Variability in tissue optical properties. - Potential for additional biological barriers in vivo that may prevent effective drug delivery and discrepancies between in vitro and in vivo results due to additional factors.
6	[34]	2018	- Ability to selectively excite different electrochemical luminophores (ECL). - Ability to detect multiple biomarkers for prostate cancer (PSA, miRNA-141, sarcosine). - Separation of the organic solvent containing the ECL reagents from the bioanalytes using the closed BPE design, enabling biological applications.	- The low water-solubility of the iridium complexes used limits their biological application. - The potential for ECL emission quenching at high concentrations of the co-reactant TPrA. - The need to optimize the concentration range of the Ru(II) complex to avoid the classic ECL emission mechanism.

**Table 2:** Literature review of treatment of skin cancer and wound healing using OLED.

S.No	Ref.	Source and Year of Publication	Advantages	Limitations
1.	[35]	2020 Wiley	- It is extremely thin, at only 6 µm, skin-like platform, making it suitable for attachable phototherapeutics. - The photonic skin has a long operating lifetime of over 100 h.	- The optimal wavelength and irradiation interval for the OLED skin were not fully determined and could be further optimized in the context of surgical wounds, and its applicability to other treatment areas may need further investigation.
2.	[36]	2023	- OLED light sources can be large-area (80 mm × 80 mm), thin, lightweight, and potentially flexible or conformable to the skin. - Inorganic LEDs have advantages like high power output, high efficiency, and low cost.	- The OLED light intensity of 2 mW/cm^2^ may not be sufficient for effectively killing and need major, and higher intensities around 10 mW/cm^2^ are needed.
				- The current OLED light sources are not bright enough.
3.	[14]	2021 Wiley	- They are intrinsically area light sources, which provide uniform illumination over the surface area of topical infections and lesions. - They have shown effectiveness in treating skin cancer, and their antimicrobial efficiency has been demonstrated for bacterial infections, suggesting their potential for treating cutaneous leishmaniosis.	- This study was only conducted in vitro, and further, in vivo studies are needed to evaluate the effectiveness of OLED-APDT for treating cutaneous leishmaniasis in actual patients. - The effectiveness of different photo-sensitizers may depend on the specific antioxidant defences of the Leishmania species, which could limit the generalizability of the findings to other photosensitizers
4.	[17]	2019	- Improved wound-healing parameters such as wound size, collagen density, neo-epidermis thickness, and number of new blood vessels, fibroblasts, and neutrophils. - Modulation of cytokine levels, in-creasing anti-inflammatory IL-1β and IL-6, and decreasing proinflammatory TNF-α.	Not mentioned.
5.	[37]	2018 Wiley	-Lightweight and thin design (0.82 g, 676 µm). - Flexible with a 20 mm bending radius. - Long operation life (>300 h). - Low-temperature operation (<40 °C). - Wide and safe application irrespective of location and time.	- Further research is needed to deter-mine the optimal wavelength of OLED. - This study only looked at in vitro Effects on fibroblasts, and further research is needed to evaluate the in vivo wound healing effects of the OLED device.

**Table 3:** Literature review of diagnosis and treatment of glioma using OLEDs.

S.No	Ref.	Source and Year of Publication	Advantages	Limitations
1.	[15]	2015	OLED: lightweight, flexible, power-efficient, compact, suitable for mPDT in small animals.	- A small sample size hinders generalization. - Need for larger sample size and extended treatment days.
2.	[65]	2024 MDPI	- Photodynamic therapy offers improved survival rates for brain tumour patients. - Minimal side effects. - Selective accumulation in cancer cells	- Insufficient accumulation of PSs in the tumour hampers PDT success. - transport to the tumour postoperative resection area.
3.	[58]	2023	- PDT induces cell death through oxida - Light dosimetry can be optimized by altering delivery geometry and timing.	- High costs and specialized equipment requirements - PS accumulation variability and reduced efficacy in hypoxic regions. - PDT efficacy is hindered by the hypoxic glioma microenvironment.
4.	[4]	2014 IEEE	- Selectively activates neurons. - Uses biocompatible plastic substrates. - Offers high-resolution emissive arrays. - Reduces power consumption significantly	- Glass substrates are rigid and not suitable for in vivo applications. - Autoclave sterilization damages OLED organic layers, affecting optical performance.
5.	[5]	2016	- Non-invasive optogenetic therapy for chronic diseases and mental health disorders. - Precision in targeting specific afferent vagus nerve branches for treatment. - Red OLED technology provides bright light for therapeutic optical stimulation.	- Existing optogenetic therapies require invasive surgery for deep brain placement. - Electrical vagus nerve stimulation devices are large and invasive. - Transcutaneous and implanted transducers lack precision for specific nerve branches.

**Table 4:** Literature review of diagnosis and treatment of breast cancer using OLEDs.

S.No	Ref.	Source Year of Publication	Advantages	Limitations
1.	[68]	2021 Elsevier	- Selective and targetable. - The use of near-infrared light in PDT allows for deeper penetration into tissue compared to other wavelengths, which could be beneficial for treating deep-seated breast tumours	- Limited light penetration depth. - The need to optimize the photosensitizer and its ability to generate cytotoxic reactive oxygen species upon light activation
2.	[23]	2023 Elsevier	- Minimally invasive design with a detectable actuator that can be easily replaced or refilled, providing convenience and flexibility - Wireless control capability for on demand delivery of drugs and light. - Partial self-powering using body motion energy, reducing the need for external power sources and increasing the device’s portability and convenience	- The device still needs further development and testing before it can be used in clinical cancer treatment and testing before it can be used in clinical cancer treatment - The current device is not fully selfpowered
3.	[74]	2021 Elsevier	- It is minimally invasive. - In vitro research on PDT can help identify optimal methods for clinical treatment. - PDT has demonstrated cytotoxic potential against breast cancer cell lines, particularly MCF-7, and the potential for enhancement through nanotechnology.	Not mentioned.

**Table 5:** Literature review of biomarkers detection using OLEDs.

S.No	Ref.	Source and Year of Publication	Advantages	Limitations
1.	[78]	2021 IEEE	- Ability to leverage existing OLED display technology to produce low-cost biosensor substrates at scale. - High diagnostic sensitivity. - Ability to combine OLED display technology with bio recognition microarray technology to create a new type of point-of-care diagnostic device.	- Cost and scalability of manufacturing the biosensor substrate.
2.	[79]	2016	- It enables efficient andcost-effective point-of-care molecular diagnostics health. - High-density, programmable, and multiplexed bio recognition in a compact and disposable configuration with clinical-level sensitivity. - Significantly reduced the cost of the biosensor substrate to just pennies persquare centimetre.	- The current approach has limitations in terms of density, programmability, and multiplexing capabilities that the authors are trying to address with their new technology. - The current approach is limited to detecting certain biomarkers.
3.	[80]	2014 IEEE	- Miniaturized design - Uses OLED and photodiode technology. - Achieves good optical performance with a bright OLED emitter and optical filters.	- Lower sensitivity compared to laboratory fluorescence-based instruments - Evaluation limited to a specific test structure with certain optical components, which may not be representative of real-world applications.
4.	[22]	2023 Elsevier	- High sensitivity and specificity for Detecting cancer biomarkers, making them desirable over traditional tech. - Low cost, high sensitivity, and high specificity of photo electrochemical techniques. - Ability to detect very low concentrations of biomarkers, which is crucial for early cancer diagnosis.	- Performance limitations of optical and photo electrochemical nanobiosensors.

**Table 6:** Literature review of diagnosis and treatment of neonatal jaundice using OLEDs.

S.No	Ref.	Source and Year of Publication	Advantages	Limitations
1.	[3]	2022 Wiley	- It can move flexibly and conform to the curvature of the human body, addressing the issue of separation from parents in traditional LED-based phototherapy. - Low voltages, addressing the disadvantages of water loss and retinal damage in traditional LED-based phototherapy.	- Limitations in terms of the wavelength and power output. - The operating reliability and temperature range of the OLED platform. - This study was limited to in vitro testing, and further research would be needed to evaluate the effectiveness of the OLED platform in actual clinical settings.
2.	[85]	2021	- Promotes more efficient bilirubin degradation, which is a key factor in the treatment of neonatal jaundice.	Not mentioned.
3.	[84]	2015	Not mentioned.	- The review considered potential harms or safety issues in addition to effectiveness.
4.	[86]	2016	-Significantly higher bilirubin decline rates in both haemolytic and non-haemolytic neonates. - Comparable rates of rebound jaundice. - Lower rates of side effects such as hyperthermia, dehydration, and skin rash.	- Small sample size of 100 neonates per group. - Higher rates of side effects (hyperthermia, dehydration, skin rash) in the fluorescent tube group compared to the LED group.
5.	[88]	2015 Wiley	- Reduced manufacturing cost. - Potential improvement in diagnostic functionality.	Not mentioned.

**Table 7:** Observation of OLEDs on various diseases.

S.No	Disease	Type of OLEDs	Wavelength	Remarks
1.	Ovarian cancer	Violet (diagnosis) NIR (treatment)	420–440 nm 700–800 nm	Triple-hole block-layer and TADF OLEDs are more efficient.
2.	Prostate cancer	Red	630–850 nm	Triple-hole block-layer and TADF OLEDs are more efficient.
3.	Cutaneous cancer and wound healing	Red	560–770 nm (wound healing) - Red wavelength (630–650 nm) for photobiomodulation - Near-infrared (NIR) wavelength (850 nm) for photodynamic therapy	Wavelength-tunable OLEDs are preferred for cancer detection and treatment.
4.	Glioma/brain cancer	Red	Range: 590–750 nm	Flexible and semitransparent OLEDs are used; more research is required in this domain.
			615–635 nm murine glioma study	
			630 nm brain tumour treatment	
5.	Mental health	Blue to red	620 nm optogenetic stimulation	More research is required.
			450–460 optimum level of activation	
6.	Breast cancer	Red to NIR	600–1100 nm	Micro-LEDs are used, which can be implemented in OLEDs for enhancement.
7.	Neonatal jaundice	Blue	460–490 nm	Flexible blue OLEDs are preferred.

**Table 8:** Various generations of OLED-PDT applications.

OLED-PDT Generations	Technology Used	Relative Cost	Relative Efficiency	Wavelength Purity
1G	Fluorescence	Low	Low	High
2G	Phosphorescence	High	High	Low
3G	Thermally activated delayed fluorescence	Low	High	Low
4G	Hyper fluorescence	Low	High	High

**Table 9:** Sensitivity and biomarker limitation.

Type of Cancer	Biomarker	Sensitivity Limit of Detection	Reference
Ovarian Cancer	CA-125	0.26 U/mL	[101]
Prostate Cancer	PSA	f-PSA-0.012 ng/mL c-PSA-0.15 ng/mL	[34]
Breast Cancer	CAE	20 nM	[66]
Lung Cancer	EGFR	1 pM	[102]
Cervical Cancer	HPV16	10 pg/mL	[78]
